# A Self-Reported Electronic Body Map Identifies Distinct Clinical Pain Phenotypes in Chronic Pancreatitis

**DOI:** 10.14309/ctg.0000000000000998

**Published:** 2026-02-20

**Authors:** Jorge D. Machicado, David Guevara-Lazo, Jonathan P. Troost, Merrick Bay, Steven E. Harte, David A. Williams, Anna S. Lok, Daniel J. Clauw

**Affiliations:** 1 Division of Gastroenterology and Hepatology, University of Michigan, Ann Arbor, Michigan, USA;; 2 Michigan Institute for Clinical & Health Research, Michigan Medicine, Ann Arbor, Michigan, USA;; 3 Chronic Pain & Fatigue Research Center, Department of Anesthesiology, University of Michigan, Ann Arbor, Michigan, USA.

**Keywords:** pancreatitis, pain mechanism, centralized pain, central nervous system, chronic pain

## Abstract

**INTRODUCTION:**

Some patients with chronic pancreatitis (CP) experience nociplastic pain due to central nervous system dysregulation, yet its recognition is limited by the lack of bedside tools. Self-reported body maps are used to assess pain widespreadedness and to differentiate pain mechanisms in chronic pain patients, but their utility in CP is unclear. This study aimed to determine whether pain widespreadedness, measured through a body map, is associated with features of nociplastic pain in CP.

**METHODS:**

We conducted a cross-sectional analysis of adults with definite CP enrolled in a single-center longitudinal cohort. Pain widespreadedness was assessed using the Michigan Body Map and categorized by the number of painful regions (0–7) and by the presence of localized vs widespread (≥3 regions) pain. Clinical features of nociplastic pain were measured using validated psychometric and pain surveys. Group comparisons were performed with multivariable regression models adjusted for demographics, comorbidities, and CP characteristics.

**RESULTS:**

Among 110 participants (mean age 54 years, 52% male), 93% reported abdominal pain. Of those with abdominal pain, 64% had widespread pain. Increasing the number of painful regions and widespread pain (vs localized) were independently associated with higher pain severity, pain interference, and neuropathic pain scores, as well as more fatigue, impaired physical functioning, greater chronic overlapping pain conditions, and poorer physical and mental health.

**DISCUSSION:**

Two-thirds of patients with CP had widespread pain assessed using a body map. This subgroup had more severe symptoms and features of nociplastic pain. This simple tool may facilitate targeted, mechanism-based pain management in CP.

## INTRODUCTION

Abdominal pain occurs in ∼90% of patients with chronic pancreatitis (CP) at some point during their disease course (1). Traditional treatment of painful CP focuses on reducing peripheral nociceptive input (e.g., tissue damage, inflammation, and pancreatic duct hypertension) and relies heavily on radiologic images (e.g., pancreatic duct stone or stricture, and pseudocyst), with up to 75% of patients receiving opioid prescriptions, ∼50% having endoscopic therapy, and ∼30% undergoing pancreatic surgery (2,3). This current approach to treatment results in half of the patients failing to achieve satisfactory pain relief and living with chronic pain for years (4).

One explanation for the refractory nature of pain in CP is that a substantial subset of patients experience what is known as “nociplastic” pain, a term coined by the International Association for the Study of Pain to describe dysregulation within the central nervous system for how pain and sensory information is processed (5). Many brain imaging studies demonstrate that some patients with CP exhibit abnormal brain processing characteristic of nociplastic pain (6–8). Nociplastic pain responds better to centrally directed therapies (e.g., exercise, mindfulness, and duloxetine) than to peripherally directed therapies (e.g., nonsteroidal anti-inflammatory drugs, and endoscopic or surgical ductal decompression) (5). Thus, identifying the involvement of nociplastic pain may enable a more tailored, mechanism-based approach to CP pain management with fewer unwarranted consequences or delays in effective care (9). However, assessing for nociplastic pain using advanced neuroimaging is costly, time-consuming, and clinically impractical.

Widespread pain throughout the body is a hallmark clinical feature of nociplastic pain (5). This can be easily assessed using a brief, 2-minute survey that includes a body map (10,11). A high number of painful regions on a body map are correlated with other hallmark symptoms of nociplastic pain, such as fatigue, unrefreshing sleep, cognitive dysfunction, negative effect, and pain interference across various pain conditions, and are associated with worse long-term health outcomes (12–15). In a study of individuals with chronic pelvic pain, those patients with widespread pain on a body map also tend to exhibit diffuse pressure pain hypersensitivity and displayed neuroimaging findings consistent with those observed in patients with fibromyalgia, the prototypical nociplastic pain condition (16,17).

To our knowledge, body maps have not previously been used in CP. We hypothesize that the number of painful regions identified on a body map in patients with CP is associated with established clinical features of nociplastic pain. If this simple tool recognizes nociplastic pain features at the point-of-care, it has the potential to facilitate a mechanism-based treatment approach in CP. Therefore, the aim of this study was to determine the association between pain widespreadedness and clinical features of nociplastic pain in CP using a state-of-the-art, comprehensive, multifactorial, self-reported pain phenotyping approach.

## MATERIALS AND METHODS

### Study design

This study is a cross-sectional analysis of an ongoing single-center, prospective, longitudinal cohort of patients with CP enrolled at the University of Michigan between June 2024 and July 2025. This study was approved by the Institutional Review Board at the University of Michigan (HUM00251979). Informed consent was provided by subjects before any study procedures. The study followed the Strengthening the Reporting of Observational Studies in Epidemiology guidelines for cross-sectional studies (18).

### Study population

Subjects aged 18–75 years with definite CP were eligible for inclusion. Definite CP was defined by the presence of pancreatic calcifications, Cambridge grade 3 or 4, or a histologic diagnosis of CP. Subjects with suspected CP or recurrent acute pancreatitis, acute pancreatitis within the past month, history of total pancreatectomy, suspected or diagnosed pancreatic cancer, active chemotherapy, serious medical or mental illnesses, cognitive impairment, or illicit drug use were excluded. The current analysis included subjects who completed baseline self-reported measures of pain and associated symptoms.

### Data collection

After enrollment, information on demographics, alcohol use, smoking, and substance use was collected by interviewing participants. Clinical data including CP diagnostic criteria, disease duration from CP diagnosis, imaging findings, presence of diabetes or exocrine pancreatic insufficiency, comorbidities for Charlson Comorbidity Index calculation, CP etiology, prior endoscopic and surgical treatments, and current medical therapies were extracted from electronic medical records. All variables were recorded using case report forms in Research Electronic Data Capture (REDCap). Each enrolled subject was provided an electronic link through text message or e-mail to complete a battery of self-reported questionnaires using Qualtrics. Electronic reminders were sent every 2 weeks up to 4 times. If questionnaires remained incomplete after these reminders, additional attempts were made to complete the questionnaires through telephone or in person during clinic visits.

### Michigan Body Map

The widespreadedness of pain was assessed using the Michigan Body Map (11,19). This self-report tool instructed subjects to check any of 35 body sites where they had experienced persistent or recurrent pain for the last 3 or more months (see Supplementary Figure 1, Supplementary Digital Content, http://links.lww.com/CTG/B480). These responses were categorized into a 7-region body map including the front trunk, back, head/neck, left arm, right arm, left leg, and right leg (see Supplementary Figure 2, Supplementary Digital Content, http://links.lww.com/CTG/B480) (13). Subjects with abdominal pain were then dichotomized into 2 groups: (i) localized pain in those who reported either abdominal pain only or abdominal pain along with pain in 1 other region (e.g., back) and (ii) widespread pain in those who experienced abdominal pain plus 2 or more other painful regions (20). Subjects without abdominal pain could be pain free or have localized (1–2 painful regions) or widespread (3 or more painful regions) extra-abdominal pain.

### Psychometric assessments

Symptoms of nociplastic pain were measured using the PROMIS 29 + 2 profile, version 2.1 (21). Physical and mental health were assessed with PROMIS Global Health version 1.2 (22). All PROMIS measures were converted to T-scores, where a score of 50 approximates the U.S. general population mean with a SD of 10. Higher scores indicate greater symptom burden for negatively worded domains (fatigue, sleep disturbance, anxiety, depression, and pain interference) and better health for positively worded domains (physical function, social roles, cognitive function, and physical/mental health). The PROMIS Preference Score (PROPr) was calculated from PROMIS-29 + 2 responses, with values ranging from −0.022 (worst health) to 1.0 (perfect health), with higher scores reflecting better health-related quality of life. The Childhood Traumatic Events Scale was administered to measure early life trauma, which has been linked to top-down nociplastic pain mechanisms (23).

### Pain assessments

Pain was evaluated using the Comprehensive Pain Assessment Tool Short Form for Chronic Pancreatitis (COMPAT-SF), a validated 6-question instrument designed specifically for CP that assesses multiple components of pancreatic pain and analgesic use within the past 3 months (24). COMPAT-SF responses yield a total pain score ranging from 0 to 100, along with subscores for pain severity, pattern, provocation, spreading, and quality. Additional pain domains were assessed using the painDETECT questionnaire for neuropathic pain features, the Pain Catastrophizing Scale for catastrophizing tendencies, and the Chronic Overlapping Pain Conditions (COPC) screener for the presence of comorbid chronic pain disorders (see Supplementary Table 1, Supplementary Digital Content, http://links.lww.com/CTG/B481).

### Statistical analysis

Demographics, clinical characteristics, and questionnaires were summarized using frequencies and percentages for categorical variables and mean ± SD for continuous variables, as appropriate. The study cohort was categorized into 5 groups according to the number of painful regions (0, 1, 2, 3, or ≥4) using the 7-region body map (14). In addition, among subjects with abdominal pain, pain was categorized as either localized or widespread. Comparisons between categories were performed using the χ^2^ test for categorical variables, and Kruskal-Wallis test or Wilcoxon rank-sum test for continuous variables. Multivariable regression models were constructed to examine the independent association of both the number of painful regions and widespread/localized phenotypes with pain and psychometric outcomes, adjusting for age, sex, Charlson Comorbidity Index, and prior endotherapy or surgery. Variables included in the multivariable analysis were selected if they had a *P*-value <0.1 in univariate analysis or were identified a priori based on their relationship with nociplastic pain. Given the role of biological sex in pain processing, an interaction term for sex as a disease modifier was included in the models (25). All models were assessed for multicollinearity. Missing item-level data were handled according to instructions provided by each instrument's manual. When individual questionnaires were missing in their entirety or could not be scored because of excessive missing items, those scores were excluded from the analysis. Statistical significance was defined as *P*-value < 0.05. Statistical analysis was conducted in SAS v9.4.

## RESULTS

### Baseline characteristics

**Figure 1.**
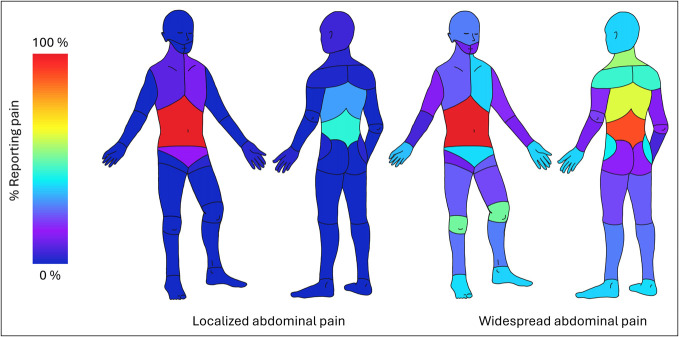
Heat body maps indicating the distribution of pain sites among patients with chronic pancreatitis and abdominal pain who have localized (1 or 2 regions) or widespread (≥3 regions) pain (n = 110).

Between June 2024 and July 2025, a total of 314 subjects were assessed for eligibility. Of these, 154 subjects with CP enrolled in the cohort. For this study, 110 subjects with definite CP who responded to baseline study questionnaires were included (see Supplementary Figure 3, Supplementary Digital Content, http://links.lww.com/CTG/B480). There were no demographic differences between respondents and nonrespondents. Using the Michigan Body Map, an average of 6.6 ± 6.2 painful sites and 3.4 ± 2 painful regions were reported (0 regions: 5; 1 region: 12; 2 regions: 26; 3 regions: 14; 4–7 regions: 53). Abdominal pain was reported by 102 (93%) subjects of which pain was widespread in 64% and localized in 36%. Figure 1 shows the proportion of painful sites in these 102 subjects according to widespread or localized distribution of pain. Among the 8 subjects without abdominal pain, 5 reported no pain and 3 had extra-abdominal pain (2 widespread and 1 localized).

Among the 110 analyzed subjects, mean age was 53.7 ± 13 years, most were White (88%), and half were male (52%). The majority had alcohol (43%) and idiopathic (34%) as the most common etiologies. Over a mean of 5.7 ± 5.3 years of disease duration, pancreatic endotherapy had been performed in 47% of subjects and pancreatic surgery in 11%. Half of the subjects were on opioids at enrollment. Subjects with 1 or more painful regions were significantly younger than those without pain (*P* = 0.01) and were more likely to have undergone endoscopic therapy (*P* = 0.04). Among the 102 subjects with abdominal pain, older age (*P* = 0.01) and higher Charlson comorbidity index (*P* = 0.02) were observed for those with widespread pain as compared with localized. Otherwise, there were no statistical differences based on the number of painful regions (Table 1) or between those with widespread and localized pain (see Supplementary Table 2, Supplementary Digital Content, http://links.lww.com/CTG/B481).

**Table 1.** Baseline characteristics

Characteristic	Overall (n = 110)	0 regions (n = 5)	1 region (n = 12)	2 regions (n = 26)	3 regions (n = 14)	4+ regions (n = 53)	*P*-value
Age, yr (mean ± SD)	53.7 ± 13.2	67.2 ± 7.2	49.8 ± 17.4	47.8 ± 13.3	51.4 ± 11.8	56.9 ± 11.3	0.01
Male sex, n (%)	57 (52)	3 (60)	8 (67)	12 (46)	5 (36)	29 (55)	0.53
White race, n (%)	97 (88)	4 (80)	10 (83)	25 (96)	12 (86)	46 (87)	0.67
Etiology, n (%)							0.54
Alcohol	47 (43)	1 (20)	3 (25)	14 (54)	6 (43)	23 (43)	
Idiopathic	37 (34)	2 (40)	5 (42)	9 (35)	6 (43)	15 (28)	
Nonalcohol	26 (24)	2 (40)	4 (33)	3 (12)	2 (14)	15 (28)	
Disease duration, yr (mean ± SD)	5.7 ± 5.3	7.4 ± 10.5	7.1 ± 7.5	5.1 ± 3.7	2.9 ± 1.9	6.4 ± 5.3	0.2
Charlson Comorbidity Index (mean ± SD)	2.6 ± 2.3	3.2 ± 1.3	1.8 ± 2.7	2.2 ± 2.1	2.4 ± 3.0	3.1 ± 2.2	0.06
Smoking status, n (%)							0.33
Current smoker	37 (34)	0 (0)	2 (17)	8 (31)	4 (29)	23 (43)	
Never smoker	40 (36)	4 (80)	6 (50)	9 (35)	6 (43)	15 (28)	
Past smoker	33 (30)	1 (20)	4 (33)	9 (35)	4 (29)	15 (28)	
History of recurrent pancreatitis, n (%)	55 (50)	1 (20)	5 (42)	14 (54)	9 (64)	26 (49)	0.49
Exocrine pancreatic insufficiency, n (%)	67 (61)	5 (100)	6 (50)	15 (58)	8 (57)	33 (62)	0.4
Diabetes mellitus, n (%)	42 (38)	2 (40)	2 (17)	12 (46)	7 (50)	19 (36)	0.4
Previous pancreatic endotherapy, n (%)	52 (47)	0 (0)	7 (58)	15 (58)	3 (21)	27 (51)	0.04
Previous celiac plexus block, n (%)	19 (17)	0 (0)	1 (8)	3 (12)	3 (21)	12 (23)	0.47
Previous pancreatic surgery, n (%)	12 (11)	0 (0)	4 (33)	1 (4)	1 (7)	6 (11)	0.08
Current antidepressant, n (%)	49 (45)	1 (20)	3 (25)	11 (44)	8 (57)	26 (49)	0.35
Current gabapentinoids, n (%)	31 (28)	0 (0)	3 (25)	6 (23)	4 (29)	18 (34)	0.52
Current opioids, n (%)	56 (51)	0 (0)	5 (42)	13 (50)	9 (64)	29 (55)	0.14

CP, chronic pancreatitis

### Association of number of painful regions with psychometric and pain outcomes

**Figure 2.**
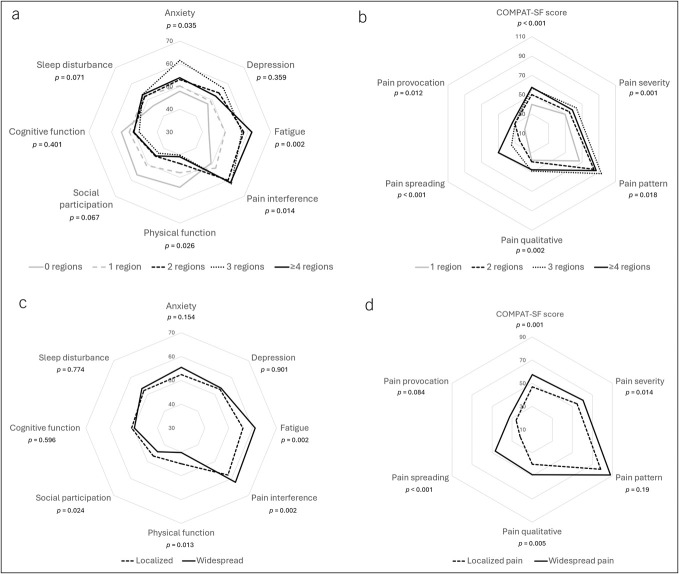
Radar charts showing (**a**) PROMIS-29+2 profile categorized by the number of painful regions, (**b**) COMPAT-SF scores categorized by the number of painful regions, (**c**) PROMIS-29+2 profile categorized by localized or widespread abdominal pain, and (**d**) COMPAT-SF scores categorized by localized or widespread abdominal pain. All *P*-values are adjusted for age, sex, Charlson Comorbidity Index, and prior endotherapy or surgery. Radar charts illustrating the PROMIS-29 symptom profile are shown for (**a**) comparisons between participants with varying numbers of painful regions (0, 1, 2, 3, or ≥4) according to the Michigan body map and (**b**) pain widespreadedness categorized as localized or widespread. The center of the plot represents a score of 30 across all 7 PROMIS-29 domains, while the outer edge represents a score of 70. Higher scores indicate worse outcomes for fatigue, sleep disturbance, pain interference, anxiety, and depression, and better outcomes for physical function, social participation, and cognitive function. Similarly, radar charts displaying COMPAT-SF subscores and total score are shown for (**c**) comparisons by the number of painful regions and (**d**) pain widespreadedness. Higher scores on any subscore indicate worse pain outcomes. The center of these plots represents a score of 10 across all COMPAT-SF domains, and the outer edge represents a score of 90. Separate lines denote distinct subgroups; lines farther from the center indicate higher scores on that domain. COMPAT-SF, Comprehensive Pain Assessment Tool Short Form; COMPAT-SF, comprehensive pain assessment tool short form; PROMIS-29+2, Patient-Reported Outcomes Measurement Information System 29+2.

**Figure 3.**
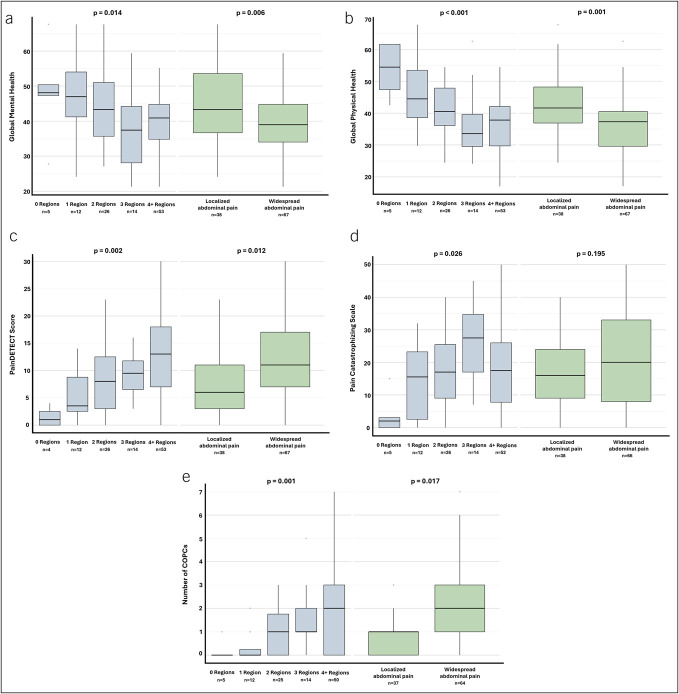
Outcomes categorized by the number of painful regions (0, 1, 2, 3, or ≥4) and by localized or widespread abdominal pain. (**a**) global mental health, (**b**) global physical health, (**c**) painDETECT, (**d**) pain catastrophizing scale, and (**e**) number of COPCs. All *P*-values are adjusted for age, sex, Charlson Comorbidity Index, and prior endotherapy or surgery. COPCs, chronic overlapping pain conditions.

Results for multivariable regression analyses, adjusted for demographics, CP characteristics, and comorbidities are given in Table 2. An increasing number of painful regions (from 0 to 4 or more) were independently associated with decreased physical function (*P* = 0.03), increased fatigue (*P* = 0.002), greater pain interference (*P* = 0.01), higher anxiety (*P* = 0.04), lower PROPr score (*P* = 0.02), reduced mental health (*P* = 0.01), and impaired physical health (*P* < 0.001; Figure 2a, Figures 3a and 3b). In addition, a higher number of painful regions were independently associated with greater scores for pain severity, pattern, provocation, spreading, quality, and total COMPAT-SF (all *P*-values <0.05, Figure 2b). Increased PainDETECT scores (*P* = 0.002), higher pain catastrophizing (*P* = 0.03), and a greater number of COPCs (*P* = 0.001) were also associated with an increasing number of painful regions in the adjusted models (Figures 3c–3e). The COPCs significantly associated with greater painful body regions included fibromyalgia, chronic low back pain, and migraines, whereas others such as irritable bowel syndrome and endometriosis were not associated (see Supplementary Table 3, Supplementary Digital Content, http://links.lww.com/CTG/B481). Neither sleep disturbance, depression, social participation, cognitive function, nor childhood trauma was significantly associated with the number of painful regions in the adjusted models (Table 2). No interactions between sex and any of the outcomes were detected.

**Table 2.** Independent associations between the number of painful regions and PROMIS 29, pancreatitis pain, and other psychosocial measures among patients with CP in multivariable analysis (n = 110)

Outcomes (mean ± SD)	0 regions (n = 5)	1 region (n = 12)	2 regions (n = 26)	3 regions (n = 14)	4+ regions (n = 53)	Adjusted *P*-value
PROMIS-292 (n = 110)						
Physical function^a^	54.3 ± 6.0	47.9 ± 8.6	43.8 ± 9.0	40.2 ± 10.1	40.9 ± 9.9	0.026
Fatigue^b^	42.8 ± 9.4	50.0 ± 13.3	58.2 ± 10.8	57.3 ± 12.8	61.7 ± 9.5	0.002
Sleep disturbances^b^	46.4 ± 11.2	52.4 ± 5.3	51.9 ± 5.7	53.3 ± 5.1	53.2 ± 5.3	0.071
Pain interference^b^	49.4 ± 10.8	52.4 ± 11.4	60.0 ± 7.5	62.1 ± 10.1	61.5 ± 10.0	0.014
Anxiety/fear^b^	47.9 ± 11.8	50.3 ± 7.9	53.0 ± 9.2	61.5 ± 12.0	53.8 ± 10.3	0.035
Depression^b^	47.5 ± 10.7	49.1 ± 8.1	54.3 ± 8.9	57.2 ± 10.2	52.3 ± 11.1	0.359
Social participation^a^	56.6 ± 8.6	50.8 ± 11.7	45.1 ± 9.1	43.2 ± 11.5	44.9 ± 10.2	0.067
Cognitive function^a^	55.7 ± 7.9	52.3 ± 9.4	50.5 ± 7.8	47.9 ± 8.7	50.3 ± 7.8	0.401
PROPr score^a^	0.6 ± 0.3	0.4 ± 0.3	0.3 ± 0.2	0.3 ± 0.3	0.3 ± 0.2	0.017
Mental health (n = 110)^a^	48.2 ± 14.1	48.4 ± 12.3	43.9 ± 11.6	38.0 ± 12.0	39.7 ± 8.0	0.014
Physical health (n = 110)^a^	53.5 ± 8.5	46.5 ± 11.6	41.2 ± 7.4	36.2 ± 10.4	37.2 ± 8.3	<0.001
COMPAT-SF (n = 98)^c^						
Pain severity^b^	12.5 ± 10.2	49.7 ± 21.0	54.7 ± 19.3	63.0 ± 24.3	58.4 ± 20.4	0.001
Pain pattern^b^	NA	66.7 ± 24.6	84.0 ± 23.8	92.9 ± 18.2	86.7 ± 22.3	0.018
Pain provocation^b^	NA	18.4 ± 13.7	30.1 ± 11.0	30.2 ± 11.9	33.0 ± 15.8	0.012
Spreading pain^b^	14.3 ± 10.5	16.7 ± 15.0	24.6 ± 12.7	34.4 ± 21.1	49.9 ± 15.5	<0.001
Qualitative pain^b^	3.3 ± 2.8	37.5 ± 21.5	39.3 ± 21.1	48.9 ± 21.2	47.5 ± 25.1	0.002
Total pain score^b^	NA	39.9 ± 12.6	50.1 ± 11.9	56.9 ± 15.1	57.5 ± 14.1	<0.001
Other measures						
No. of COPCs (n = 105)^b^	0.2 ± 0.5	0.3 ± 0.7	1.0 ± 1.0	1.6 ± 1.4	2.0 ± 1.8	0.001
Pain catastrophizing (n = 109)^b^	4.0 ± 6.3	14.0 ± 12.3	17.8 ± 9.8	26.2 ± 12.7	18.8 ± 13.7	0.026
PainDETECT (n = 109)^b^	1.5 ± 1.9	5.5 ± 4.9	8.4 ± 6.4	9.7 ± 4.1	12.6 ± 8.0	0.002
Childhood trauma (n = 110)^b^	1.8 ± 4.0	6.3 ± 10.4	7.9 ± 7.6	11.2 ± 10.4	7.4 ± 7.6	0.622

COMPAT-SF, Comprehensive Pain Assessment Tool Short Form; COPCs, chronic overlapping pain conditions; PROMIS-29+2, Patient-Reported Outcomes Measurement Information System 29+2; PROPr, PROMIS Preference Score.

a A higher score on this score indicates better outcome.

b A higher score on this score indicates worse outcome.

c Included only subjects in whom a total COMPAT-SF pain score could be calculated. To calculate a total pain score, at least 4 of the 5 subscores need to be calculated and the pain severity subscore needs to be one of these 4. A minimum number of responses are needed to calculate each of the subscores as detailed in the score manual. In patients without abdominal pain (n = 8), a total pain score could not be calculated.

### Association of widespread or localized abdominal pain with psychometric and pain outcomes

Results for multivariable regression analyses comparing localized versus widespread pain in patients with CP and abdominal pain (*P* = 102) are shown in Table 3. Widespread pain was independently associated with lower physical function (*P* = 0.01), greater fatigue (*P* = 0.002), increased pain interference (*P* = 0.002), poorer social participation (*P* = 0.02), lower PROPr score (*P* = 0.03), reduced mental health (*P* = 0.006), and impaired physical health (*P* < 0.001; Figure 2c, Figures 3a and 3b). Widespread pain was independently associated with higher scores of pain severity, spreading, quality, and total COMPAT-SF (all *P*-values <0.05, Figure 2b), but not with pain pattern and provocation. Higher PainDETECT scores (*P* = 0.01) and a greater number of COPCs (*P* = 0.02) were associated with widespread pain in adjusted models (Figures 3c–3e). Specific COPCs associated with widespread pain included fibromyalgia, temporomandibular disorder, chronic low back pain, and migraines, whereas other COPCs were not different between widespread and localized pain (see Supplementary Table 3, Supplementary Digital Content, http://links.lww.com/CTG/B481). Scores for sleep disturbance, anxiety, depression, cognitive function, pain catastrophizing, and childhood trauma were not associated with widespread pain in multivariable analysis (Table 3). A significant interaction was found between sex and fatigue—the effect of widespread pain on fatigue was an additional 8.9 points lower for women compared with men. No other sex interactions were observed for the outcomes.

**Table 3.** Independent associations of localized and widespread pain phenotypes with PROMIS 29, pancreatitis pain, and other psychosocial measures among patients with CP and abdominal pain in multivariable analysis (n = 102)

Outcomes (mean ± SD)	Localized (n = 37)	Widespread (n = 65)	Adjusted mean difference in score (95% CI)	Adjusted *P*-value	**R^2^**
PROMIS-29+2 (n = 102)					
Physical function^a^	45.0 ± 9.1	40.3 ± 9.5	−6.86 (−12.22, −1.50)	0.013	0.272
Fatigue^b^	55.9 ± 12.2	61.1 ± 10.2	10.27 (3.98, 16.57)	0.002	0.319
Sleep disturbances^b^	52.2 ± 5.5	53.4 ± 5.2	0.44 (−2.58, 3.46)	0.774	0.232
Pain interference^b^	57.8 ± 9.5	62.2 ± 9.4	8.99 (3.44, 14.54)	0.002	0.259
Anxiety/fear^b^	52.4 ± 8.7	55.5 ± 11.2	4.38 (−1.67, 10.44)	0.154	0.187
Depression^b^	53.0 ± 8.8	53.7 ± 11.0	−0.38 (−6.47, 5.70)	0.901	0.144
Social participation^a^	46.6 ± 10.2	44.0 ± 9.9	−6.80 (−12.66, −0.93)	0.024	0.218
Cognitive function^a^	50.8 ± 8.2	49.6 ± 7.9	−1.31 (−6.19, 3.58)	0.596	0.094
PROPr score^a^	0.3 ± 0.2	0.3 ± 0.2	−0.13 (−0.26, −0.01)	0.034	0.267
Mental health (n = 102)^a^	45.1 ± 11.9	39.1 ± 8.8	−8.23 (−13.99, −2.47)	0.006	0.255
Physical health (n = 102)^a^	42.7 ± 9.2	36.6 ± 8.4	−8.92 (−13.88, −3.97)	0.001	0.390
COMPAT-SF (n = 98)^c^					
Pain severity^b^	54.7 ± 17.8	60.8 ± 19.8	13.87 (2.84, 24.90)	0.014	0.348
Pain pattern^b^	78.4 ± 25.1	88.1 ± 21.5	9.23 (−4.66, 23.13)	0.190	0.180
Pain provocation^b^	26.1 ± 13.1	32.4 ± 15.0	7.56 (−1.03, 16.16)	0.084	0.220
Spreading pain^b^	22.0 ± 14.0	47.0 ± 18.0	24.50 (14.19, 34.80)	<0.001	0.487
Qualitative pain^b^	39.9 ± 20.3	49.0 ± 23.5	19.91 (6.22, 33.60)	0.005	0.343
Total pain score^b^	46.9 ± 12.8	57.4 ± 14.2	13.77 (5.68, 21.85)	0.001	0.313
Other measures					
Number of COPCs (n = 97)^b^	0.8 ± 0.9	2.0 ± 1.7	1.11 (0.20, 2.02)	0.017	0.247
Pain catastrophizing (n = 101)^b^	16.6 ± 10.8	21.0 ± 13.5	4.97 (−2.59, 12.53)	0.195	0.196
PainDETECT (n = 102)^b^	7.6 ± 6.0	12.2 ± 7.4	5.27 (1.17, 9.37)	0.012	0.268
Childhood trauma (n = 102)^b^	7.4 ± 8.6	8.3 ± 8.4	1.93 (−2.95, 6.82)	0.434	0.167

COMPAT-SF, Comprehensive Pain Assessment Tool Short Form; COPCs, chronic overlapping pain conditions; PROMIS-29+2, Patient-Reported Outcomes Measurement Information System 29+2; PROPr, PROMIS Preference Score.

a A higher score on this score indicates better outcome.

b A higher score on this score indicates worse outcome.

c Included only subjects in whom a total COMPAT-SF pain score could be calculated. To calculate a total pain score, at least 4 of the 5 subscores need to be calculated and the pain severity subscore needs to be one of these 4. A minimum number of responses are needed to calculate each of the subscores as detailed in the score manual.

## DISCUSSION

In clinical practice, pain in CP is often attributed to nociceptive mechanisms, such as tissue damage, inflammation, or pancreatic duct hypertension. Using a body map to quantify the spatial extent of pain, we found that most patients with painful CP reported widespread pain, a hallmark of nociplastic pain. Increased pain widespreadedness, measured either by the number of painful regions or by categorizing pain as localized versus widespread, was independently associated with (i) measures of pain severity, interference, and neuropathic features; (ii) other characteristics of nociplastic pain such as fatigue, impaired physical functioning, and COPCs; and (iii) poor physical and mental health. These findings introduce a novel and practical approach for phenotyping pain in CP (i.e., localized pain translates nociceptive/peripheral pain, while widespread pain translates nociplastic/central pain), with important implications for improving pain assessments and tailoring therapeutic interventions.

The central mechanisms of pain have been studied in patients with CP using a validated quantitative sensory testing (QST) protocol that includes repetitive pinprick, pressure stimulation, and a cold-water test (26). Using this QST, a phenotype of widespread hyperalgesia is associated with increased fatigue, lower physical function, poorer global health, and higher pain intensity/interference and is considered a marker of nociplastic pain (27). Despite proving objective phenotypes, clinical applicability of QST in CP is limited by the need for experienced operators, specialized equipment, prolonged testing duration (15–20 minutes), and considerable interindividual and intraindividual variability. In this study, we demonstrated that a simple, 2-minute, self-reported electronic body map provides associations with health outcomes comparable with pancreatic QST. We observed a dose-response relationship between the number of painful regions and both declining health status and increasing pain severity. These associations were independent of demographic and CP clinical features, such as age, sex, disease duration, and prior endoscopic or surgical interventions. Similar findings have been reported in other chronic pain conditions using different body maps including the 2011 Fibromyalgia Survey Criteria, the Michigan Body Map, and the Collaborative Health Outcomes Information Registry body map (13,14,28,29).

In addition to demonstrating that body maps help distinguish the health experiences of patients with CP, our study further showed that among patients with abdominal pain, localized and widespread pain represent distinct clinical phenotypes. The widespread pain group displayed more constitutional symptoms (physical function, pain, social, and energy disturbances) and a greater number of COPCs, both of which are prominent features of nociplastic pain and resemble the widespread hyperalgesia phenotype with QST (30). Importantly, these differences were independent of psychiatric comorbidities, such as depression, anxiety, and childhood trauma, and persisted after adjusting for several demographic and clinical features. Although our findings support a possible role of nociplastic pain mechanisms in patients with CP and widespread pain, we cannot confirm this given the current lack of standardized diagnostic criteria for nociplastic pain and the nonspecificity of symptom burden. However, extensive neurobiological studies support the relationship of widespread pain and nociplastic pain in other chronic pain conditions (e.g., chronic pelvic pain and knee/hip osteoarthritis), showing that patients with widespread pain exhibit altered brain structure and function, as well as enhanced sensitivity to experimental pain, which are hallmarks of nociplastic pain (16,17,31). Even if widespread pain is not diagnostic of nociplastic pain in CP, our results suggest that body maps can serve as a useful screening tool to identify patients who may benefit from further evaluation for COPCs and related negative outcomes.

Using widespread pain as a surrogate marker for nociplastic pain (32), we found that the prevalence of nociplastic pain in our cohort ranged from 48% to 61%, depending on whether a cutoff of painful regions of ≥3 or ≥4 was used. Although previous studies have not directly estimated the prevalence of nociplastic pain in CP, randomized trials have reported refractory pain in more than 50% of patients after endoscopic or surgical drainage in large duct CP, a figure that is comparable with our estimates of nociplastic pain (33,34). This high burden of nociplastic pain in CP may explain why, in a recent randomized trial of patients with painful CP and an obstructed pancreatic duct, endoscopic drainage using extracorporeal shockwave lithotripsy and endoscopic retrograde cholangiopancreatography (ERCP) was not superior to a sham intervention at 24 weeks (35). Addressing only ductal obstruction or nociceptive input does not resolve pain in patients with nociplastic pain and may put them at unwarranted risk for procedural complications. A simple body map could potentially guide more rational management of painful CP, by detecting the mechanism of pain before starting any treatment and predicting treatment response based on the underlying mechanism of pain. Theoretically, patients with widespread pain may be more likely to benefit from nonpharmacologic interventions (e.g., exercise, cognitive behavioral therapy, and yoga), antidepressants (e.g., duloxetine and amitriptyline), and neuromodulation approaches (e.g., gabapentinoids, transcranial magnetic stimulation, and acupuncture), and less likely to benefit from peripherally directed therapies (e.g., ERCP and pancreatic surgery) (9). Such a precision medicine approach using body maps has been shown to guide pain management in other pain disorders and may support a personalized care pathway in painful CP (20,36–38). Future studies are needed to demonstrate the usefulness of body maps to predict response to pain therapies in CP (e.g., ERCP, surgery, and centrally active therapies). If accurate, this would be a simple way to predict treatment response to pain therapies at the point-of-care and could be modeled by other gastrointestinal conditions.

Our study has some limitations. This was a single-center study conducted at a US tertiary care center and included predominantly White individuals, which may limit the generalizability of our findings. However, our cohort had baseline characteristics similar to other published US cohorts, including the ongoing PROCEED Consortium (39). While the study cohort was prospectively collected and is undergoing longitudinal follow-up, the present analysis was cross-sectional, precluding the determination of causality. Although it is not possible to determine whether nociplastic features preceded the development of widespread pain, the value of our findings lies in the practicality of the body map to detect nociplastic pain at the bedside in <2 minutes. As the study relied on electronic questionnaires to assess pain, there is a risk of selection bias favoring participation of subjects with pain and technological literacy. The study used self-reported outcomes, which does not address the etiology of patient's pain and carries a risk of recall and misclassification biases. There were some missing data; specifically, COMPAT-SF scores could not be calculated for 8 subjects with painless CP and 4 additional subjects who did not complete their questionnaires. All other surveys had more than 95% completion rates, with both the Michigan Body Map and PROMIS-29 completed by all participants.

The electronic Michigan Body Map used in this study has shown reliability and construct validity in chronic pain populations (11,19); however, this is its first application in CP and therefore requires future validation. Simply counting the number of painful sites (0–35 in the Michigan Body Map) may overestimate pain widespreadedness due to contiguous spread of peripheral nociceptive pain (e.g., pancreatic pain affecting the abdomen, pelvis, groins, chest, upper back, and lower back) (40). Thus, we summarized painful sites into 7 regions in which patients with nociceptive pancreatic pain could report pain in the front trunk (abdomen, pelvis, groins, and chest) and/or back (upper/lower back, hips, and buttocks). To address potential ceiling effects with ≥4 painful regions, a single category was used for this scenario as in previous studies (13,14). Finally, we categorized body map results as localized or widespread pain, defining widespread pain as ≥3 painful regions based on previous definitions (12,13,20). Subjects reporting pain in 2 regions were included in the localized category because pancreatic pain may radiate to the back.

In conclusion, a simple body map enables clinicians to differentiate between widespread and localized pain subgroups in CP, which represent distinct phenotypes with different psychometric profiles and pain outcomes. The use of body maps may assist clinicians in distinguishing between pain mechanisms and recognizing nociplastic pain at the bedside. Future research should explore the role of body maps in predicting response to various therapies and their potential utility in guiding personalized treatment decisions. In addition, longitudinal analyses of this cohort will provide valuable insights into the natural history of pain phenotypes using body maps and other self-reported tools in CP.

## CONFLICTS OF INTEREST

**Guarantor of the article:** Jorge D. Machicado, MD, MPH.

**Specific author contributions:** Study concept and design: J.D.M., A.S.L., D.J.C. Data collection and investigation: D.G.L., M.B. Statistical analysis: J.P.T. Drafting of the manuscript: J.D.M. Generation of data: D.G.L., M.B. Data interpretation, final approval of the manuscript: all authors.

**Financial support:** Research reported in this publication was supported by the American College of Gastroenterology Junior Faculty Career Development Award (J.D.M.) and by the Robert A. Winn Excellence in Clinical Trials Career Development Award (J.D.M.). Analytic support was provided in part by the National Center for Advancing Translational Sciences (JPT, UM1TR004404).

**Potential competing interests:** None to report.

**Transparency statement:** Deidentified data, analytic methods, and study materials can be made available on request to the corresponding author.

**IRB approval statement:** This study was approved by the Institutional Review Board at the University of Michigan (HUM00251979). Informed consent was provided by subjects before any study procedures.

**Study Highlights**

**WHAT IS KNOWN**

✓ Pain in chronic pancreatitis (CP) is common, difficult to treat, and caused by different pathophysiologic mechanisms.
✓ Differentiating nociplastic/centralized pain from other mechanisms requires sophisticated methods and simple bedside tools are not available.
✓ Widespread pain is a hallmark of nociplastic pain that may distinguish pain mechanisms using a simple body map.

**WHAT IS NEW HERE**

✓ Two-thirds of patients with CP show widespread pain on a body map, likely of nociplastic origin.
✓ Widespread pain in CP is independently associated with worse symptoms, physical function, and quality of life.
✓ Body maps may enable mechanism-based pain assessment and more personalized treatments of pain in CP.

## Supplementary Material

## ABBREVIATIONS:

COMPAT-SF: Comprehensive Pain Assessment Tool Short Form for Chronic Pancreatitis
COPC: chronic overlapping pain condition
CP: chronic pancreatitis
PROMIS PROPr: PROMIS Preference Score
PROMIS: Patient-Reported Outcomes Measurement Information System
QST: quantitative sensory testing
