# Supplementary material for: A Self-Reported Electronic Body Map Identifies Distinct Clinical Pain Phenotypes in Chronic Pancreatitis
Source: Clin Transl Gastroenterol. 2026 Feb 20;17(5):e00998. doi: 10.14309/ctg.0000000000000998 (PMC13193298; doi:10.14309/ctg.0000000000000998)

Supplementary Figure 1: Image of the electronic Michigan Body Map.

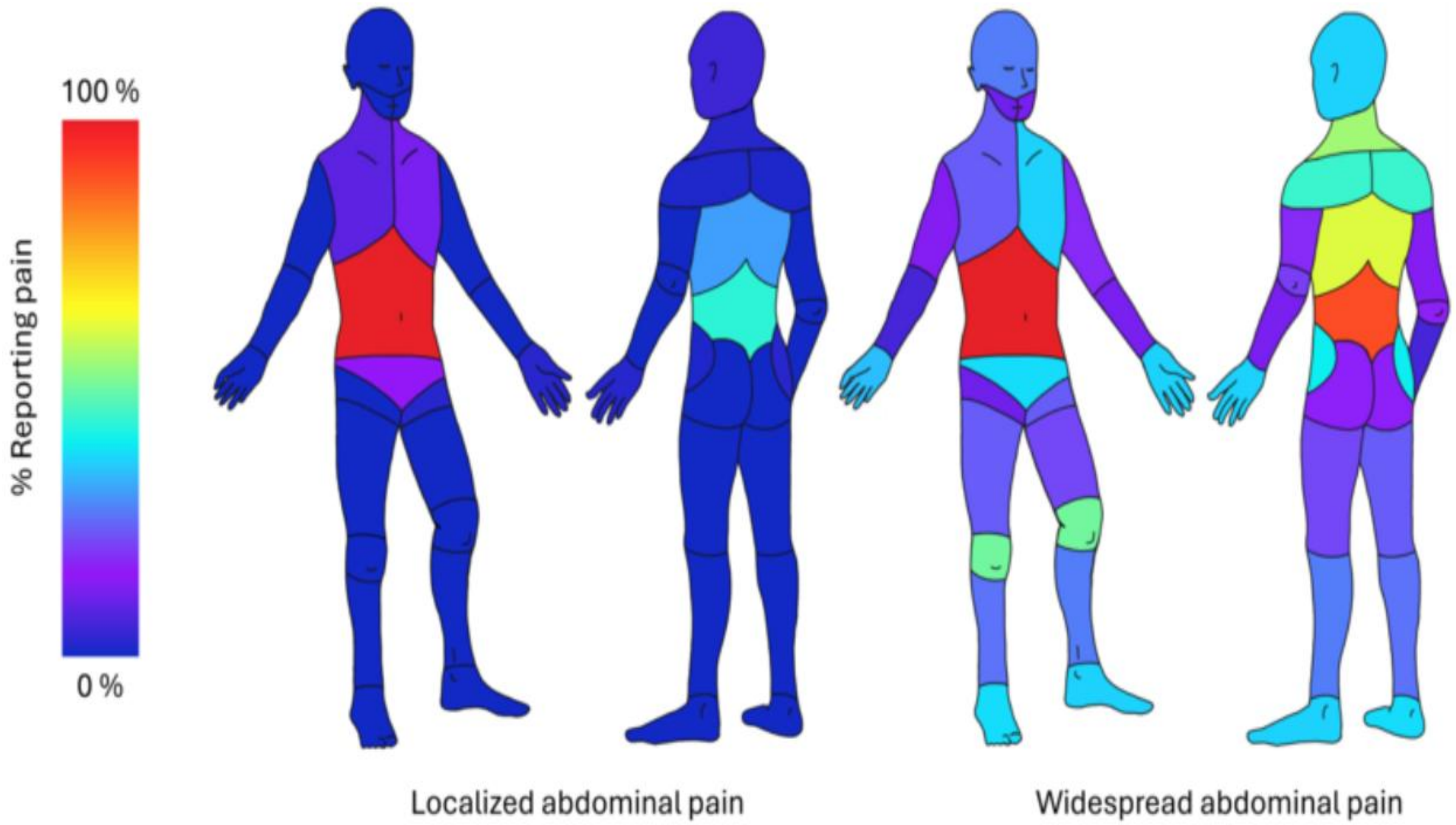

Supplementary Figure 2: Pain group assignments into seven painful regions using the Michigan Body Map.

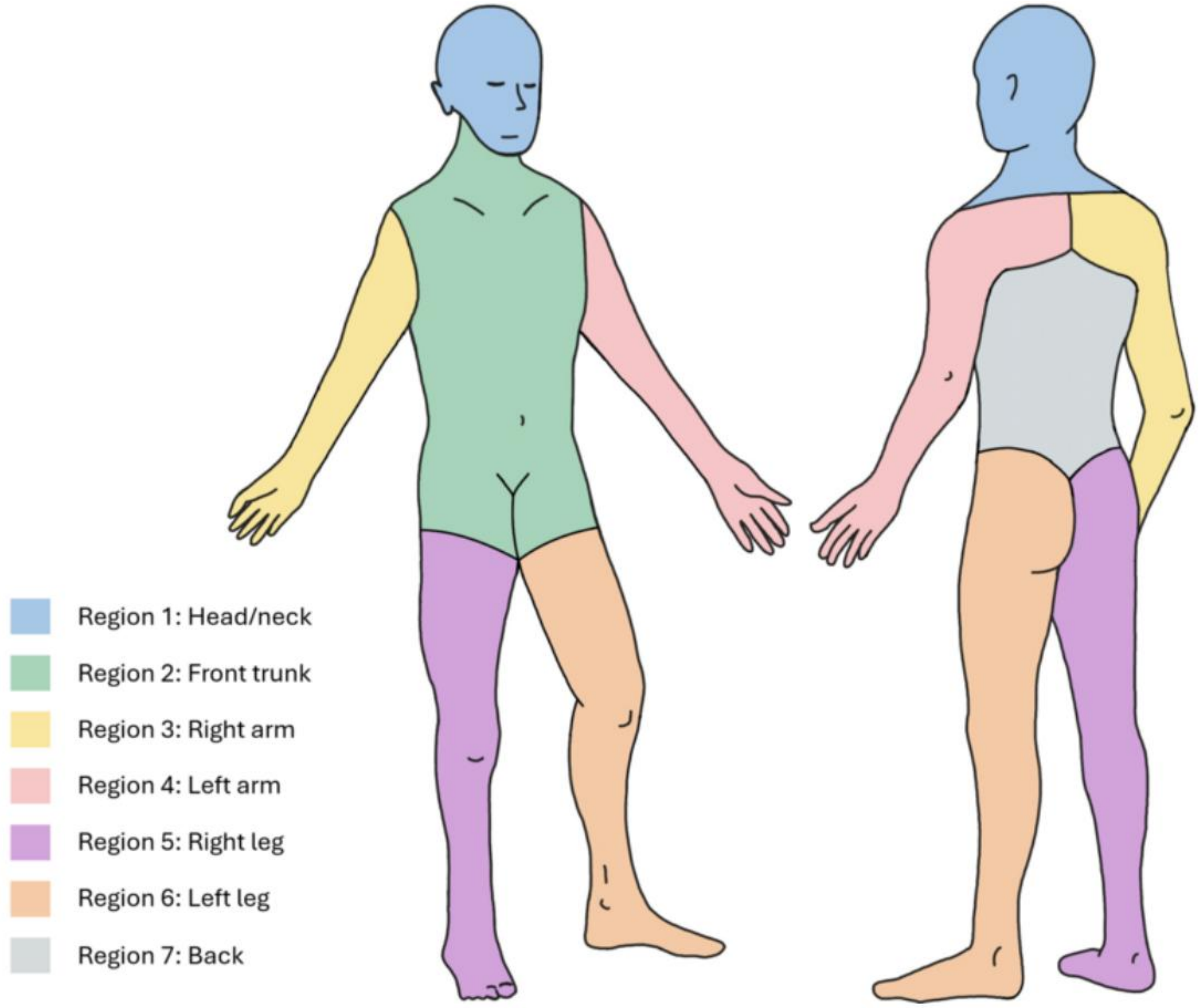

Supplementary Figure 3: Study flowchart displaying participant recruitment and pain distribution categories.

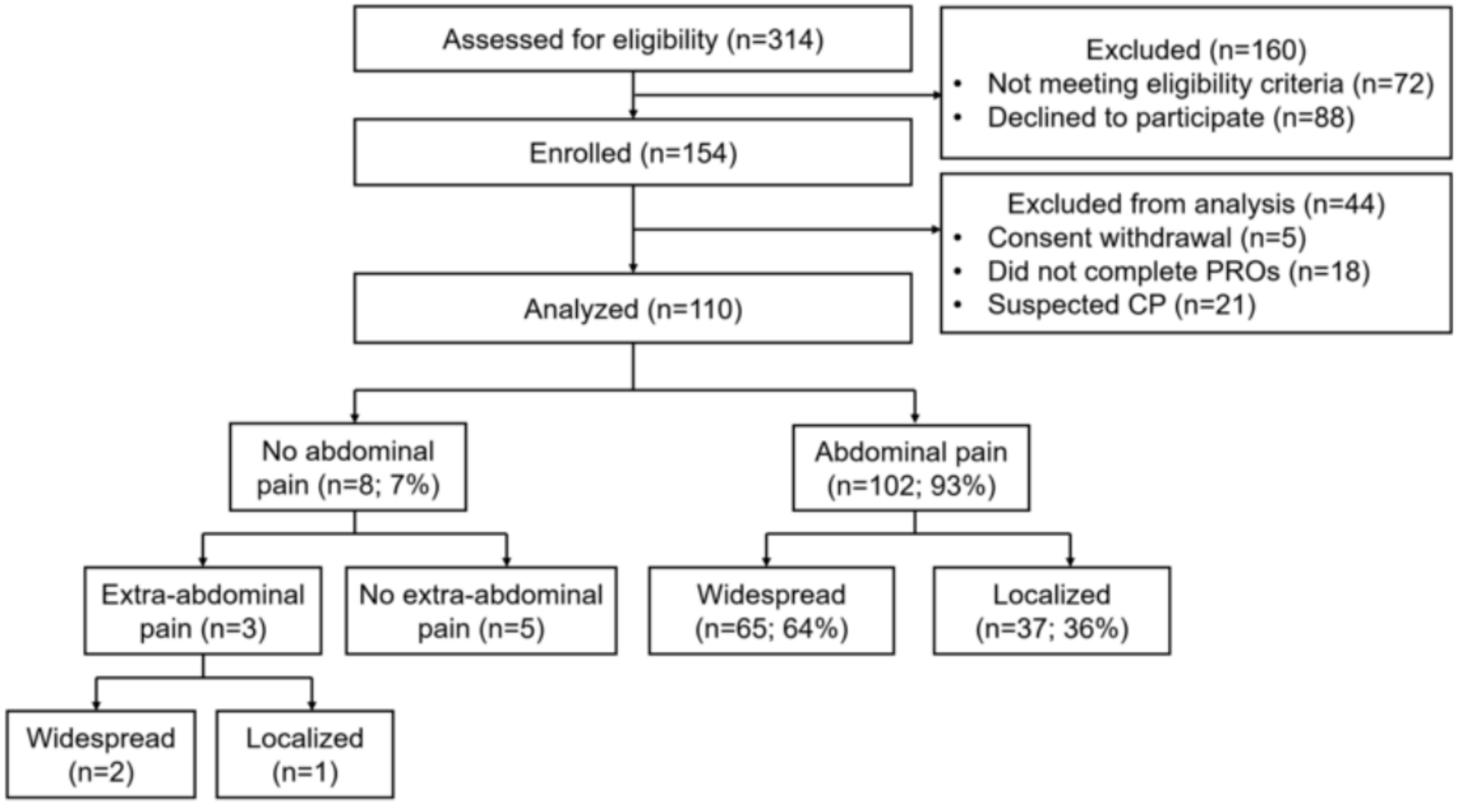

Supplement: Supplementary file 1 [file ct9-17-e00998-s001.pdf]
