# Supplementary material for: A Self-Reported Electronic Body Map Identifies Distinct Clinical Pain Phenotypes in Chronic Pancreatitis
Source: Clin Transl Gastroenterol. 2026 Feb 20;17(5):e00998. doi: 10.14309/ctg.0000000000000998 (PMC13193298; doi:10.14309/ctg.0000000000000998)
Supplement: Supplementary file 2 [file ct9-17-e00998-s002.docx]

**Supplementary Table 1**: Summary of questionnaires used for pain, psychosocial, and health assessment

| Questionnaires | Domains assessed | Scoring and interpretation | Comments |
| --- | --- | --- | --- |
| PROMIS-29+2 v2.1 | Pain interference, fatigue, sleep disturbance, depression, anxiety, physical function, cognitive function, social role | Domain-specific T-scores (mean=50, SD=10) standardized to US population. Higher scores indicate more fatigue, sleep disturbance, anxiety, depression and pain interference, and better physical function, social roles, and cognitive function. | 31-item instrument that evaluates 7 health domains in the past 7 days. The PROPr score is a single measure derived from these 7 domains and that summarizes health status, with values ranging from -0.022 (worst health) to 1.0 (perfect health). |
| PROMIS Global Health v1.2 | Overall physical and mental health | Physical and Mental Health T-scores (mean=50, SD=10) standardized to US population. Higher scores reflect better health. | 10-item global health measure that assesses quality of life. |
| painDETECT | Neuropathic pain | Score ranges from –1 to 38, with higher scores representing greater likelihood of neuropathic pain. | 9-item screening tool to detect descriptors of neuropathic pain. |
| COMPAT-SF (*Comprehensive Pain Assessment Tool Short Form*) | Pain severity, pattern, provocation, spreading, and quality | Each domain receives sub-scores that range from 0 to 100. A total pain score that ranges from 0 to 100 is calculated if at least 4 domains can be calculated, of where one has to be the severity dimension score. Higher scores denote greater pain. | 6-question CP-specific tool to evaluate pain over the past 3 months. Pain severity includes pain intensity and analgesic use. Pain pattern can be constant (score 100) or intermittent (score 50). Pain quality measures descriptors (e.g. cramping, shooting, stabbing). Pain provocation refers to pain triggers (e.g. food, stress, exercise). |
| Pain Catastrophizing Scale | Rumination, magnification, and helplessness | Score range from 0 to 52, with higher score indicating greater catastrophizing. | 6-item validated measure for catastrophizing, which refers to the tendency to think about pain as being awful, extremely unpleasant, and superlatively disruptive. |
| COPC Screener (*Chronic Overlapping Pain Conditions Screener*) | Fibromyalgia, IBS, TMD, vulvodynia, ME/CFS, IC/BPS, chronic prostatitis, endometriosis, migraine/TTH, cLBP | Score ranges from 0 to 10, with higher score representing more pain conditions. Each COPC represents 1 point. | Multi-item validated tool to rapidly assess for the presence of 10 common COPCs through self-report. |
| Childhood Traumatic Events Scale | Early life trauma events (death of a close friend or family member, parental separation, sexual abuse, physical abuse, serious illness or injury) | Score ranges from 0 to 42, with higher score implying more childhood trauma. | 6-item questionnaire that screens for the presence and severity of traumatic events before age 17. |

PROMIS: Patient-Reported Outcomes Measurement Information System; SD: standard deviation; PROPr: PROMIS-preference score; CP: chronic pancreatitis; IBS: irritable bowel syndrome; TMD: temporomandibular disorder; ME/CFS: myalgic encephalomyelitis / chronic fatigue syndrome; IC/BPS: interstitial cystitis / bladder pain syndrome; TTH: tension-type headache; cLBP: chronic low back pain

**Supplement Table 2:** Baseline characteristics stratified by localized or widespread pain

| **Characteristic** | **Overall (n=102)** | **Localized (n=37)** | **Widespread (n=65)** | **p-value** |
| --- | --- | --- | --- | --- |
| Age, years (mean ± SD) | 52.7 ± 13.0 | 47.9 ± 14.3 | 55.4 ± 11.5 | 0.010 |
| Male sex, n (%) | 51 (50) | 19 (51) | 32 (49) | 0.840 |
| White race, n (%) | 90 (88) | 34 (92) | 56 (86) | 0.390 |
| Etiology, n (%) |  |  |  | 0.670 |
| Alcohol | 46 (45) | 17 (46) | 29 (45) |  |
| Idiopathic | 32 (31) | 13 (35) | 19 (29) |  |
| Other | 24 (24) | 7 (19) | 17 (26) |  |
| Disease duration, years (mean ± SD) | 5.7 ± 5.1 | 5.9 ± 5.2 | 5.7 ± 5.0 | 0.890 |
| Charlson Comorbidity Index (mean ± SD) | 2.6 ± 2.4 | 2.0 ± 2.3 | 3.0 ± 2.4 | 0.020 |
| Smoking status, n (%) |  |  |  | 0.340 |
| Current smoker | 37 (36) | 10 (27) | 27 (42) |  |
| Past smoker | 33 (32) | 14 (38) | 19 (29) |  |
| Never smoker | 32 (31) | 13 (35) | 19 (29) |  |
| History of recurrent pancreatitis, n (%) | 54 (53) | 19 (51) | 35 (54) | 0.810 |
| Exocrine pancreatic insufficiency, n (%) | 60 (59) | 20 (54) | 40 (62) | 0.460 |
| Diabetes mellitus, n (%) | 39 (38) | 13 (35) | 26 (40) | 0.630 |
| Previous pancreatic endotherapy, n (%) | 52 (51) | 22 (59) | 30 (46) | 0.200 |
| Previous celiac plexus block, n (%) | 19 (19) | 4 (11) | 15 (23) | 0.130 |
| Previous pancreatic surgery, n (%) | 12 (12) | 5 (14) | 7 (11) | 0.680 |
| Current antidepressant, n (%) | 48 (48) | 14 (39) | 34 (52) | 0.200 |
| Current gabapentinoids, n (%) | 31 (30) | 9 (24) | 22 (34) | 0.310 |
| Current opioids, n (%) | 56 (55) | 18 (49) | 38 (58) | 0.340 |

CP: Chronic pancreatitis; SD: Standard deviation

**Supplementary Table 3:** Association of number of painful body regions and widespread/localized pain phenotypes with COPCs (univariable analysis)

| **COPCs, n (%)** | **0 regions (n=5)** | **1 region (n=12)** | **2 regions (n=25)** | **3 regions (n=14)** | **4+ regions (n=49)** | **p-value** | **Localized (n=36)** | **Widespread (n=61)** | **p-value** |
| --- | --- | --- | --- | --- | --- | --- | --- | --- | --- |
| Fibromyalgia | 0 (0) | 0 (0) | 1 (4) | 0 (0) | 17 (35) | <0.001 | 1 (3) | 17 (28) | 0.002 |
| Irritable Bowel syndrome | 0 (0) | 2 (18) | 11 (44) | 7 (50) | 19 (39) | 0.19 | 13 (37) | 26 (43) | 0.6 |
| Temporomandibular Joint Disorder | 0 (0) | 0 (0) | 0 (0) | 1 (7) | 8 (16) | 0.1 | 0 (0) | 9 (15) | 0.02 |
| Myalgic Encephalomyelitis/ Chronic Fatigue Syndrome | 0 (0) | 1 (8) | 5 (20) | 4 (29) | 5 (10) | 0.3 | 6 (17) | 9 (15) | 0.8 |
| Chronic Low Back Pain | 0 (0) | 0 (0) | 7 (28) | 3 (21) | 30 (61) | <0.001 | 7 (19) | 33 (54) | 0.001 |
| Urologic Chronic Pelvic Pain Syndrome | 1 (20) | 1 (8) | 1 (4) | 4 (29) | 7 (14) | 0.27 | 2 (6) | 11 (18) | 0.08 |
| Endometriosis | 0 (0) | 0 (0) | 0 (0) | 1 (7) | 3 (6) | 0.59 | 0 (0) | 4 (7) | 0.12 |
| Migraine | 0 (0) | 0 (0) | 0 (0) | 2 (14) | 11 (22) | 0.03 | 0 (0) | 13 (21) | 0.003 |
| Tension Headache | 0 (0) | 0 (0) | 0 (0) | 0 (0) | 2 (4) | 0.68 | 0 (0) | 2 (3) | 0.27 |
| Vulvodynia/Vulvar Pain Disorder | 0 (0) | 0 (0) | 0 (0) | 0 (0) | 0 (0) | NA | 0 (0) | 0 (0) | NA |

COPCs: chronic overlapping pain conditions
